# Supplementary material for: An integrated bioinformatics platform for investigating the human E3 ubiquitin ligase-substrate interaction network
Source: Nat Commun. 2017 Aug 24;8:347. doi: 10.1038/s41467-017-00299-9 (PMC5570908; doi:10.1038/s41467-017-00299-9)
Supplement: Supplementary file 1 — Supplementary Information [file 41467_2017_299_MOESM1_ESM.pdf]

File name: Supplementary Information
Description: Supplementary figures, supplementary table, supplementary methods and supplementary references.

File name: Supplementary Data 1
Description: E3-substrate interactions dataset together with the supporting literature information

File name: Peer review file
Description:

Supplementary Methods

E3 recognition consensus motif

E3s may associate with the specific substrate by recognizing a short liner sequence motif. For each E3 in GSP, we predict its recognition consensus motif based on two parallel sequence datasets: one is the sequence data of this E3's substrates in GSP (target dataset) to build the motif, and the other is that of all the proteins interacting with this E3 in GSP (background dataset) for background probability calculations. The reference sequences of human proteome were downloaded from Swiss-Prot (version: May 2013) and the protein interaction datasets from HPRD[1].

Step 1: To build the motif from protein sequences, we firstly define a kind of amino acid as central. Then the target and background datasets are converted into position-weight matrices (target matrix and background matrix) of equal dimensions by counting frequency of all residues at the six positions upstream and downstream of the selected central amino acid. Then the hypergeometric probability matrix is calculated using these two matrices, where the element of the matrix is the *P*-value calculated using the hypergeometric distribution test for residues *x* at position *j*.

Step 2: We identified the highly correlated residue/position pairs with the lowest *P*-values. The *P*-values of residue/position pairs must be lower than a probability threshold ($P<10^{-3}$ in our algorithm) and occurrence must be higher than a certain threshold (2 in our algorithm). After founding such a pair, both the target and background matrixes are reduced by removing sequences do not contain this residue/position pair. Then a new hypergeometric probability matrix is calculated using the new target and background matrices and a new residue/position pair is identified. This process repeats until no more residue/position pairs satisfying constraint are detected. Then the first motif will be the tally of residue/position pairs selected during this step and the its confidence score is defined as the sum of *log(1/P)*.

Step 3: To identify other motifs, step 3 is implemented to reduce the target and background matrices by removing the sequences matching the motif identifying by step 2. Then step 1 and 2 are repeated to find new motifs until no more significant motifs can be identified.

Finally, we assign another kind of center amino acids and repeat the previous three steps until all 20 types of amino acids are used as the center amino acid.

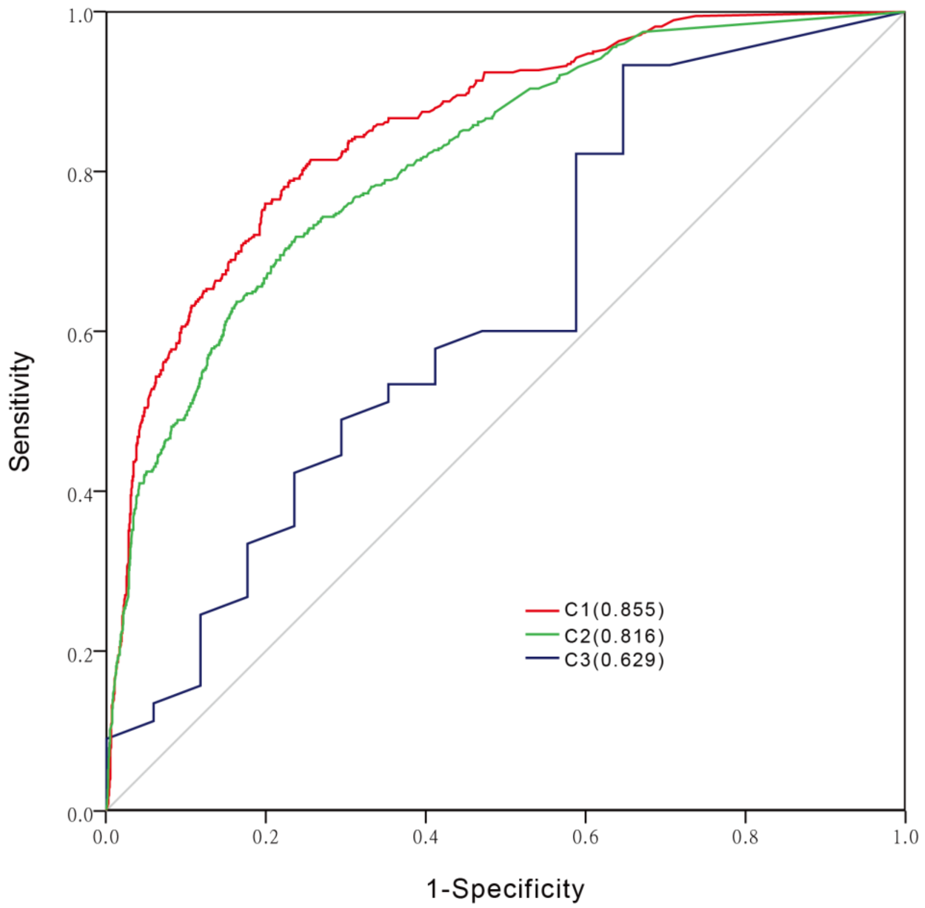

Supplementary Figure 1 ROC curves for UbiBrowser according to the protocol of Park and Marcotte against the test data sets C1, C2 and C3. C1 (Both E3s and substrates in the test set can be found in the training set), C2 (Either E3s or substrates in the test set can be found in the training set) and C3 (Neither E3s nor substrates in the test set can be found in the training set). Each point on the ROC curves corresponds to sensitivity and specificity against a particular likelihood ratio cutoff. Different test datasets corresponding to these curves are labeled in the legends. The numbers in parentheses refer to the AUC under ROC curves for different test datasets.

Figure 6

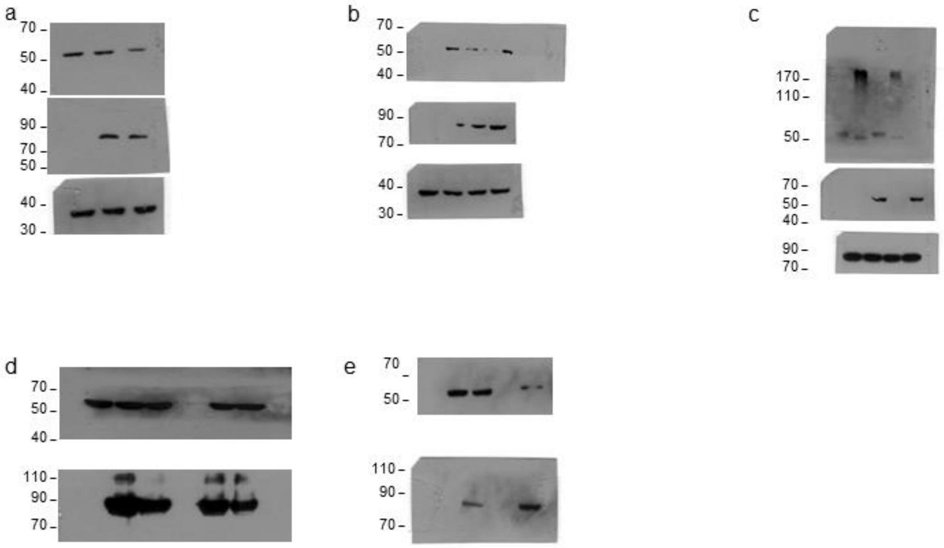

Supplementary Figure 2. Uncropped scans for Western blot data in main figures.

Supplementary Table 1 manual curation to reveal the difference between UbiBrowser and Kai-Yao Huang et al.'s work

Manual curation for 100 ESIs randomly sampling from UbiBrowser.

E3	Predicted substrate	Relation between E3 and predicted substrate in literature	Reference
ITCH	BID	Substrate	PMID: 20392206
STUB1	HIF1A	Substrate	PMID: 23880665
NEDD4L	KCNQ3	Substrate	Arévalo J C. Journal of Receptor, Ligand and Channel Research, 2015, 8: 53-63.
SMURF1	AXIN1	Substrate	PMID: 23959799
MDM2	NFATC3	Regulator	PMID: 26795951
RLIM	TP53	Regulator	PMID: 23650532

Manual curation for 100 ESIs randomly sampling from Kai-Yao Huang et al.

E3	Predicted substrate	Relation between E3 and predicted substrate in literature.	Reference
CUL4A	TSC2	Substrate	PMID: 18381890
RNF168	H2AFX	Substrate	PMID: 26507658

NEDD4L	SCN5A	Substrate	Minegishi S, Ishigami T, Ushio H, et al. Journal of Hypertension, 2010, 28: e351.
MDM2	CSNK1A1	Regulator	PMID: 19759023
TRAF3	WDR5	Regulator	PMID: 26519536
RBX1	CAND1	Regulator	PMID: 18805092
BRCA1	E2F4	Regulator	PMID: 20133863
IRAK4	MYD88	Regulator	PMID: 20485341
CUL4A	WDR76	Regulator	PMID: 17588513
DDB1	UBR5	Regulator	PMID: 24028781
FBXL6	SKP1	Regulator	Zhou W. Biochemical Characterization of the Estrogen Receptor Alpha (ERα) Coactivator E6-AP, SKP2 and FBXL6[J]. 2014.
NFX1	SIN3A	Regulator	PMID: 18505829
CUL5	LRRC41	Regulator	PMID: 22709582
CUL4A	DCAF10	Regulator	PMID: 17588513
BARD1	UIMC1	Regulator	PMID: 20096808
SIAH2	PIAS1	Regulator	PMID: 17987106
TRIM27	UBE2D1	Regulator	PMID: 22829933
ANAPC5	CDC16	Regulator	PMID: 24163370
ITCH	NDFIP2	Regulator	PMID: 20534535
DTX1	ITCH	Regulator	PMID: 18727490
MDM2	TAF1	Regulator	PMID: 17237821
RBX1	CKS1B	Regulator	PMID: 26549015
SKP2	CDC34	Regulator	PMID: 10375532
FBXO6	SKP1	Regulator	PMID: 22268729

Supplementary References:

1. Keshava Prasad, T. S. *et al.* Human Protein Reference Database--2009 update. *Nucleic Acids Res.* **37,** D767–772 (2009).
